# Assessment of immune response to a lyophilized peste-des-petits-ruminants virus vaccine in three different breeds of goats

**DOI:** 10.14202/vetworld.2016.568-571

**Published:** 2016-06-08

**Authors:** S. S. Begum, G. Mahato, P. Sharma, M. Hussain, A. Saleque

**Affiliations:** 1 Research Associate, ICAR-NRC on Yak, Dirang - 790 101, Arunachal Pradesh, India; 2 Department of Veterinary Epidemiology and Preventive Medicine, College of Veterinary Science, Assam Agricultural University, Khanapara Campus, Guwahati - 781 022, Assam, India; 3 Virologist, North East Regional Disease Diagnostic Laboratory, Khanapara, Guwahati - 781 022, Assam, India; 4 Senior Technical Officer, ICAR-NRC on Yak, Dirang - 790 101, Arunachal Pradesh, India; 5 Chief Scientist, Goat Research Station, Assam Agricultural University, Burnihat, Kamrup - 793 101, Assam, India

**Keywords:** goat, immune, peste-des-petits-ruminants, vaccine

## Abstract

**Aim:**

Immune response to a lyophilized peste-des-petits-ruminants virus (PPRV) vaccine was evaluated in three different breeds of goats.

**Materials and Methods:**

Three breeds of goats consisting six number of animals in three groups, i.e., Group A (local Assam hill goat), Group B (cross-bred), and Group C (Beetal goats) were randomly selected for evaluating the immune response to a lyophilized PPRV vaccine.

**Results:**

A higher rise in the overall mean serum antibody titer was observed in Group A (40.50±3.74) than in Group B (37.58±37.58) and Group C (35.90±3.29) during the study period.

**Conclusion:**

Initially, a negative PPRV specific serum antibody titer was recorded in all the groups at 0^th^ day of vaccination. Serum antibody titer in the vaccinated goats started rising gradually from the 14^th^ day post vaccination. Later higher rise in the overall mean serum antibody titer in Group A (local Assam hill goat) lead to the conclusion that higher serum antibody titer in local non-descript breed might be due to their better adaptation to the environmental condition.

## Introduction

Peste-des-petits-ruminants (PPR) is an important contagious viral disease of goats and sheep, often associated with high morbidity and mortality and was first reported in sheep and goats, in 1942, in Côte d’Ivoire, West Africa. It is emerging in new regions of the world, causing significant economic losses [1] and threatens the food security and sustainable livelihood of farmers across Africa, the Middle East, and Asia [2]. Although presence of PPR-like disease has been suspected earlier in a retrospective study [3], its presence was confirmed in India, in 1987, from Arasur village of Villupuram district of Tamil Nadu [4].

The disease causes more severe lesions in goats than sheep [5], and in India, the disease occurs round the year with the maximum outbreaks reported during the winter and rainy seasons. Therefore, vaccination just before the onset of rainy/winter season is considered as an appropriate step for the control of the disease. Of late, three groups of scientists have developed vero cell line based live attenuated PPR vaccines around the world using the different lineage of PPR virus (PPRV) of either goat or sheep origin for prophylaxis of disease [6-9]. Studies have been carried out to identify the sources of variation and also to unravel the genetic variance in the PPRV vaccine elicited immune response in goat kids [10]. Such studies have revealed significant variability for response to vaccination, which may be due to significant sources of variation such as environmental determinants, cohort, age at vaccination, and maternal environment. Study of comparative immunogenicity of two PPR vaccines in South Indian sheep and goats under field conditions gave no significant difference between the two strains, revealing that the two vaccine strains are equally efficacious [11].

Currently, PPR outbreaks are being reported regularly from different parts of the country [12-17], and there are very few reports on the prevalence of the disease in Assam. Furthermore, since vaccination is the mainstay for the control of PPR for goats in India, keeping these points in view, the present study was undertaken to assess the immune response of a lyophilized PPRV vaccine.

## Materials and Methods

### Ethical approval

Ethical approval for the study was obtained from Institutional Animal ethics Committee of College of Veterinary Science, Assam Agricultural University, Khanapara, Guwahati-781022, Assam.

### Management profiles

The goats were maintained under semi-intensive system of management. The floors of the animal houses were either build with concrete cement or wooden planks and were cleaned regularly with broom and water by the concerned persons, and no bedding material was provided in any of the herd. Common feeding troughs were used for feed and water. Artificial insemination or natural breeding with selected bucks was done in the farm.

### Selection of animal

Relevant data regarding age, sex, and previous history of infection were recorded for all the animals to ascertain the significance of these factors with positive cases of PPRV infection. During the study, apparently healthy goats were selected for vaccination. The status of parasitic infestation was evaluated on the basis of fecal examination and history of deworming whenever required, the animals were dewormed with Fentas plus @ 1 tablet per 10-30 kg body weight orally 10 days before vaccination.

### Reference vaccine

The lyophilized PPRV vaccine was procured from Raksha-PPR, Indian Immunologicals (batch no.01PPR29/11).

### Vaccination of animals

The vaccine was reconstituted with 100 ml of the diluent (provided with the vaccine) to prepare 100 doses, and then, it was administered subcutaneously at 1 ml per goat. The vaccinated goats were monitored for 48 h after primary vaccination, and no changes in the body temperature or any other clinical signs were observed.

### Collection of serum samples

Serum samples from the vaccinated animals were collected at 0, 14, 30, 45, 60, and 90 days of post-vaccination for assay of PPRV specific antibody titer.

Assay of PPRV specific antibody titer was done using PPR c-ELISA kit for PPRV antibody detection that was obtained from Rinderpest Laboratory, Division of Virology, IVRI, Mukteswar.

### Statistical analysis

Statistical analysis for assay of PPRV antibody was done using Statistical Package for Social Sciences, version 14.

## Results and Discussion

Initially, a negative PPRV specific serum antibody titer was recorded in all the groups at 0^th^ day of vaccination. Serum antibody titer in the vaccinated goats started rising gradually, and a higher rise in the overall mean serum antibody titer was observed in Group A (40.50±3.74) than in Group B (37.58±37.58) and Group C (35.90±3.29) during the study period (Table-1).

**Table-1:** Mean±SE of serum antibody titer of vaccinated and control group of goats.

GROUP no.	0 day	14 days	30 days	45 days	60 days	90 days	Overall mean
A (Local)	*	27.07±8.56^a^_A_	51.27±0.13^b^_A_	52.68±0.12^b^_A_	54.78±0.41^b^_A_	57.20±0.78^b^_A_	40.50±3.74^a^
B (Cross bred)	*	40.55±0.27^a^_B_	42.29±0.62^ab^_B_	43.96±0.60^ac^_B_	48.04±1.38^bc^_A_	50.65±1.50^c^_AB_	37.58±37.58^b^
C (Beetal)	*	27.41±8.68^a^_A_	43.95±1.15^b^_B_	45.58±1.12^b^_A_	48.72±0.76^b^_A_	49.76±0.69^b^_B_	35.90±3.29^b^
D (Control)	*	*	*	*	*	*	*
Overall mean	*	23.76±4.19^a^	34.38±4.21^b^	35.56±4.34^b^	37.89±4.61^bc^	39.40±4.80^c^	

* No antibody titer. Means bearing same subscript does not differ significantly in a row. Means bearing same superscript does not differ significantly in a column.

SE=Standard error

From the present study, it was observed that higher serum antibody titer in local non-descript breed might be due to their relatively high resistance to disease and better adaptation to the environmental condition. The influence of stress factor in antibody production has also been observed, and it has been recorded that stress can inhibit the development of lymphocyte response to antigen, e.g. the response to a vaccine [18]. Exotic animals might have suffered from adaptability stress, and thereby, this may hamper insufficient production of antibody following immunization for which there was lower antibody titer in comparison to local non-descript and crossbred goats. Attenuated *Morbillivirus* vaccines also induce cell-mediated immunity [19], which may be important for protection. It is not clear which immune effectors (systemic neutralizing antibodies, cytotoxic T-cell, or mucosal immunity) can be correlated with protection following vaccination with the PPRV vaccine, but antibodies are most likely to be involved because the passive transfer of immunity via colostrum may provide protection [20,21].

The control group did not show any antibody titer to the vaccine throughout the study period, and statistical analysis showed a highly significant difference (p<0.01) of antibody titer between the groups, days of vaccination, and within the groups and days (Table-2).

**Table-2:** Analysis of variance for variate titer.

Source of variation	df	Sum of square	Mean sum of square	F value
Animal group	3	39366.28	13122.09	332.04**
Days	5	27024.44	5404.89	136.76**
Animal group×Day	15	10222.80	681.519	17.25**
Error	120	4742.34	39.52	

** p<0.01

Apart from the importation of animals from disease-free region, vaccination with the commercially available attenuated vaccine is one of the best measures for prevention of the disease. A number of attenuated vaccines in freeze-dried form, recombinant subunit vaccine, DNA vaccine, etc., are commercially available now-a-days for vaccinating the animals against PPR. Current vaccination schedules require the immunization of susceptible animal at least every 3 years [22,23], and vaccination in animals of 4-6 months age is recommended [24].

Animals vaccinated with an attenuated PPR vaccine are unable to transmit the challenge virus to animals with which they are in contact [25]. Furthermore, vaccinated animals produce high amount of neutralizing antibodies against the H, F, and F proteins similar to those recovered from a natural infection [26,27]. In a report, a single immunization with PPR vaccine conferred solid protection in sheep and goats for 3 years [28].

## Conclusion

Based on the present study, we conclude that local breed of goats showed a higher immune response to Raksha-PPR vaccine (Indian immunological) than crossbred and Beetal breeds. Although live attenuated vaccines are able to induce both humoral and cell-mediated immune response and to keep long-term neutralizing antibodies against PPRVs at a high level, a potential possibility in the reversion of vaccine strains to virulence, albeit unreported so far, should not be neglected [29]. Availability of a marker vaccine (for differentiation of vaccinated animals and infected animals by use of an appropriate diagnostic tool) against PPR along with associated diagnostic tools may ease the PPR eradication program both in India and also at the global level [30].

## Authors’ Contributions

The present study was a part of SSB’s original research work during Ph.D. thesis program. GM and PS had designed the plan of work. SSB and MH carried out the experiment in the farm, and SSB carried out the laboratory work. SSB, GM, PS, and AS analyzed the results. All the authors read and approved the final manuscript.
